# Trophic Factors in Inflammation and Regeneration: The Role of MANF and CDNF

**DOI:** 10.3389/fphys.2018.01629

**Published:** 2018-11-20

**Authors:** Pedro Sousa-Victor, Heinrich Jasper, Joana Neves

**Affiliations:** ^1^Paul F. Glenn Center for Biology of Aging Research, Buck Institute for Research on Aging, Novato, CA, United States; ^2^Immunology Discovery, Genentech, Inc., South San Francisco, CA, United States

**Keywords:** inflammation, regeneration, MANF, CDNF, immune modulation

## Abstract

Regeneration is an important process in multicellular organisms, responsible for homeostatic renewal and repair of different organs after injury. Immune cell activation is observed at early stages of the regenerative response and its regulation is essential for regenerative success. Thus, immune regulators play central roles in optimizing regenerative responses. Neurotrophic factors (NTFs) are secreted molecules, defined by their ability to support neuronal cell types. However, emerging evidence suggests that they can also play important functions in the regulation of immune cell activation and tissue repair. Here we discuss the literature supporting a role of NTFs in the regulation of inflammation and regeneration. We will focus, in particular, in the emerging roles of mesencephalic astrocyte-derived neurotrophic factor (MANF) and cerebral dopamine neurotrophic factor (CDNF) in the regulation of immune cell function and in the central role that immune modulation plays in their biological activity *in vivo*. Finally, we will discuss the potential use of these factors to optimize regenerative success *in vivo*, both within and beyond the nervous system.

## Introduction

Inflammatory and regenerative responses are tightly co-regulated during tissue repair (Aurora and Olson, 2014). Deregulation of inflammatory pathways in aging and disease imposes a critical limitation to the ability of tissues to repair after injuries and molecules with the ability to limit inflammation are good candidates to improve regeneration in humans (Neves et al., 2017). Recent literature suggests that neurotrophic factors (NTFs), a class of molecules broadly defined by their ability to support neuronal growth, survival and differentiation, are also important regulators of inflammation and repair with potential application in regenerative medicine.

There are two main families of NTFs: neurotrophins, including Brain-derived neurotrophic factor (BDNF), Nerve growth factor (NGF), Neurotrophin-3 (NT-3) and Neurotrophin-4 (NT-4); and glial cell-line derived neurotrophic factor family ligands (GFLs), of which Glial cell line-derived neurotrophic factor (GDNF) is the most studied member (Airaksinen et al., 1999; Chao, 2003). Neurotrophins exert their trophic effects by signaling through tropomyosin-receptor kinase (Trk) receptor tyrosine kinases or by p75 neurotrophin receptor (p75NTR), a receptor that is also shared by immune regulators, such as tumor necrosis factor (TNF) family cytokines (Chao, 2003). GFLs, on the other hand, signal through the transmembrane Ret receptor tyrosine kinase, and receptor activation is mediated by GDNF family receptor alpha-1 (GFRα1) (Airaksinen et al., 1999).Interestingly, these receptors also have functions outside the nervous system, including in hematopoietic stem cells and immune cells (Fonseca-Pereira et al., 2014; Ibiza et al., 2016). A different class of molecules, referred to as neuropoietic cytokines, was initially described by its role in the regulation of immune response, but has since been found to play important trophic role in the nervous system (Bauer et al., 2007). Thus, mounting evidence points to the existence of common pathways in the regulation of inflammation and neuroprotection.

More recently, a new class of evolutionarily conserved NTFs has been discovered, comprising two members in mammals – mesencephalic astrocyte-derived neurotrophic factor (MANF) and cerebral dopamine neurotrophic factor (CDNF), with one single ortholog in invertebrates, MANF. MANF and CDNF were initially discovered by their neuroprotective activities in dopaminergic neurons *in vitro* and *in vivo* (Petrova et al., 2003; Lindholm et al., 2007; Voutilainen et al., 2009) and thus classified as NTFs. However, these molecules are structurally distinct from all other classes of NTFs, signal through a yet unknown receptor/mechanism (Lindahl et al., 2017) and their physiological activity extends beyond the nervous system (Tadimalla et al., 2008; Glembotski et al., 2012; Lindahl et al., 2014; Chen et al., 2015; Liu et al., 2018). Moreover, similarly, to what has been found for neuropoietic cytokines, MANF and CDNF also play important roles in the regulation of immune responses (Zhao et al., 2014; Chen et al., 2015; Neves et al., 2016) and this immune modulatory function is essential for their biological activity *in vivo*, promoting neuroprotection and regenerative success (Neves et al., 2016).

Cytoprotection and inflammation are processes that need to be precisely coordinated during regenerative responses and, thus, molecules that act at the intersection between these processes are good candidates to act as regulators of regeneration *in vivo*. Evidence suggests that classical NTFs can indeed play such role in the regulation of regeneration, both through regulation of immune function and direct actions on stem cells. Here, we will discuss the literature supporting a role for NTFs in the regulation of inflammation and regeneration. We will focus in particular in the emerging functions of MANF and CDNF and speculate on their potential to act as pro-repair factors in regenerative systems beyond the nervous system.

## Inflammation and Regeneration

Tissue regeneration requires the precise coordination of a multi-staged immune response with the activation and differentiation of progenitor cells for the efficient repair of damaged organs (reviewed in Aurora and Olson, 2014). Macrophages play a crucial role in the biphasic activation of the immune system at injury sites, and are necessary for the controlled progression of the regenerative process (Arnold et al., 2007; Nahrendorf et al., 2007; Lucas et al., 2010; Miron et al., 2013). In all systems, the early phase of the regenerative response is dominated by pro-inflammatory (M1-like) macrophages, which participate in debris clearance and stem cell activation. Subsequently, pro-repair macrophages (M2-like) coordinate cell differentiation and tissue reconstruction. In multiple systems, deregulation of this timely transition from pro-inflammatory to pro-repair cytokine production compromises regenerative success (Arnold et al., 2007; Lucas et al., 2010; Perdiguero et al., 2011). Beyond macrophages, T regulatory cells (Tregs) are also important regulators of tissue repair by modulating inflammatory signaling in different systems (Lei et al., 2015).

## NTFs in Immune Cell Signaling

Since the early identification of members of the neurotrophin family acting on immune cells (Vega et al., 2003), a role for NTFs in the cross-talk between nervous and immune systems has been suggested (Kerschensteiner et al., 2003) and more recently demonstrated in the context of CNS injury and brain pathologies.

Similarly, to other regenerative paradigms, damage to the CNS involves the activation and mobilization of microglia (the resident immune cells of the brain) and macrophages (recruited from circulation), followed by the coordinated balance of pro-inflammatory (M1) and pro-repair (M2) microglial phenotypes (Jin and Yamashita, 2016). M2-type macrophages promote remyelination (Miron et al., 2013) and repair after spinal cord injury (SCI) (Shechter et al., 2009), optic nerve injury (London et al., 2011), and stroke (Wattananit et al., 2016).

In the nervous system, microglial cells, and macrophages express several neurotrophin family members and their respective receptors, functioning both as sources and targets of NTFs. Neurotrophins were shown to impact microglial function at different levels: BDNF stimulates microglial proliferation (Elkabes et al., 1996; Zhang et al., 2003), while NT-3 can stimulate phagocytic activity of microglial cells *in vitro* (Elkabes et al., 1996). In mouse models of SCI, BDNF delivery can increase the proportion of M2 macrophages, while inhibiting the expression of pro-inflammatory cytokines at injury sites (Ji et al., 2015). NGF can also act directly in microglial cells by promoting chemotactic migratory activity, potentially contributing to recruitment of additional immune cells at injury sites (De Simone et al., 2007). Findings that macrophages and microglia are some of the main p75NTR-expressing cells in multiple sclerosis (MS) lesions (Dowling et al., 1999), together with evidence that NGF/p75 activation can limit the microglia’s inflammatory cascade (Neumann et al., 1998), also seem to implicate NGF signaling in neurotrophin-mediated immune modulation at MS lesion sites. Recently, the immune modulatory role of NGF was further supported by evidence showing that, in the context of Alzheimer’s disease-related insults, NGF signaling can blunt the pro-inflammatory state of microglia and promote a neuroprotective and pro-repair microenvironment (Rizzi et al., 2018).

Outside the CNS, macrophages also express and respond to neurotrophins (Barouch et al., 2001; Samah et al., 2008; Williams et al., 2015). BDNF has been associated with inflammation and injury in the aging heart (Cai et al., 2006), while NGF has been implicated in the inflammatory response following myocardial damage (Govoni et al., 2011), a process where innate immune cells also coordinate the inflammatory response (Ong et al., 2018). Given the above described studies implicating neurotrophins in microglia’s immune modulation at injury sites, it will be important to explore whether these or other NTFs can play a role in coordinating the immune response during regeneration outside the CNS.

Age-related changes in NTF levels have been reported in humans and roedent models and decreased levels of different NTFs have been associated age-related disease in the CNS (Tapia-Arancibia et al., 2008; Budni et al., 2015). In contrast, in the heart, age-associated increased levels of BDNF are associated with worse outcomes after injury (Cai et al., 2006). Future studies will be required to determine the relative contribution of each tissue to the age-related changes in NTFs and the consequences of these changes for immune cell regulation.

## MANF and CDNF in Immune Cell Signaling

Although MANF and CDNF were initially discovered by their neurotrophic activities, further studies revealed that MANF and CDNF are highly expressed in non-neural tissues and that their cytoprotective activity extends beyond the dopaminergic system (Tadimalla et al., 2008, Airavaara et al., 2009; Glembotski et al., 2012; Lindahl et al., 2014; Yang et al., 2014; Neves et al., 2016; Gao et al., 2017; Liu et al., 2018; Lu et al., 2018; Matlik et al., 2018). Moreover, MANF is found in circulation in the blood and it has been associated with several non-neuronal diseases in humans, including inflammatory diseases (Wang et al., 2014; Chen et al., 2015; Yavarna et al., 2015; Galli et al., 2016), suggesting a role beyond neuroprotection. Consistently, recent data suggest that cytoprotection accounts only partially for MANF and CDNF’s biological activity. We and others found that MANF and CDNF can also act directly on immune cells and modulate their inflammatory phenotype by reducing pro-inflammatory signaling and promoting pro-reparative activation of macrophages (Zhao et al., 2014; Chen et al., 2015; Neves et al., 2016) and that this function is required for the protective function observed *in vivo* (Neves et al., 2016).

### MANF and Immune Cell Signaling

Although it was initially discovered as an astrocyte-derived factor, MANF is also highly expressed in several immune cell types of sponges, flies and mammals, including humans (Chen et al., 2015; Liu et al., 2015; Neves et al., 2016; Sereno et al., 2017), and it is dynamically regulated in these cell types in response to damage and inflammatory signals (Chen et al., 2015; Neves et al., 2016; Sereno et al., 2017). While the mechanism involved in MANF-mediated regulation of inflammation is still largely unexplored, all studies published to date support the notion that MANF stimulation or MANF expression in immune cells results in the inhibition of pro-inflammatory signaling.

We found that in response to retinal damage in flies and mice, the expression of MANF is transiently induced in immune cells (hemocytes in flies and macrophages in mice) and this induction is necessary to promote recovery from damage and repair (Neves et al., 2016). Surprisingly, this function is not associated with a direct cytoprotective activity on retinal cells by MANF secreted from immune cells. Instead, the tissue repair promoting activity of immune cell-derived MANF relies on an autocrine function that regulates a phenotypic switch of macrophages (Neves et al., 2016). Using *in vitro* assays on hemocytes isolated from *Drosophila*, or macrophages differentiated from bone marrow precursors from mice, we showed that both cell types respond to stimulation with recombinant MANF protein, activating a phenotypic switch and transcriptional profile associated with pro-reparative functions of macrophages. Importantly this phenotypic switch was also observed *in vivo* and mediated the tissue repair functions associated with MANF overexpression or delivery: In flies, overexpression of MANF on hemocytes that are unable to respond to MANF signaling through inactivation of KDELR – a receptor required for MANF binding at the cell surface (Henderson et al., 2013) – failed to promote retinal repair after injury. Similarly, the protective effects of MANF delivery against retinal damage were lost in mouse models where macrophages were ablated or MANF-irresponsive (Neves et al., 2016). This was the first demonstration in an *in vivo* context that the cytoprotective function of MANF is, at least in part, mediated by immune modulation.

The ability of MANF to regulate immune signaling was also observed in other contexts, not directly associated with tissue damage. In a rabbit model of antigen-induced arthritis, MANF was found up-regulated in synovial tissues associated with inflammatory cell infiltration. MANF was particularly induced in fibroblast-like synoviocytes (FLSs), one of the main inflammatory cell types in the synovium proposed to trigger synovial inflammation, an important contributor to disease progression. In FLSs, MANF function was associated with the negative regulation of inflammatory signaling and the inhibition of the proliferative expansion of inflammatory FLSs (Chen et al., 2015). Mechanistically, this study proposes that the function of MANF in FLSs relies on its inhibitory interactions with p65 in the nucleus, which prevents p65 binding to DNA and consequent activation of NFκB target genes, including several pro-inflammatory cytokines (TNFα, Il-8, and IL-1β) (Chen et al., 2015). Although this study provides an interesting insight into the mechanism by which MANF may regulate inflammatory signaling, it is important to note that MANF is mostly found in the cytoplasm and reports of its localization in the nucleus are rare. Thus, while such mechanism may operate in acute events of inflammatory signaling it is unlikely that it represents the main mechanism of action for MANF in immune cells. The physiological consequence of the inhibitory effect of MANF on inflammatory signaling for disease progression also remains to be explored.

Interestingly, the ability of MANF to inhibit inflammatory signaling is not restricted to cells of the immune system and it has also been reported in astrocytes subject to oxygen–glucose deprivation (Zhao et al., 2013) and neural stem cells (NSCs) exposed to LPS, a potent pro-inflammatory stimulus (Zhu et al., 2016). In both cases, the effects were also associated with the negative regulation of NFκB signaling and consequent inhibition of pro-inflammatory cytokines. In NSCs this was further associated with inhibition of p38 signaling without affecting other MAPK pathways (JNK and ERK). Although these studies provide links between several signaling pathways and the effects of MANF on inflammatory signaling they do not demonstrate a mechanistic link between them, since no inhibitors of the pathways are used to demonstrate their requirement for the effects of MANF.

**FIGURE 1:**
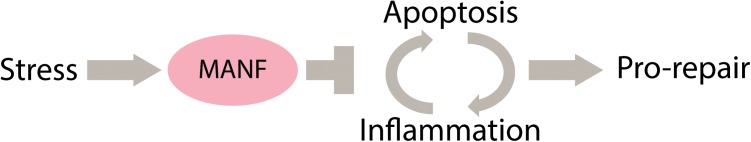
Proposed model for the function of MANF in tissue repair through a synergistic activity as an inhibitor of apoptosis and inflammation.

Thus, MANF has evolutionarily conserved immune regulatory functions that contribute to the negative regulation of inflammation. During chronic injuries, apoptosis and inflammation can create a positive auto-regulatory loop that promotes further tissue damage. Thus, *in vivo*, the cytoprotective and immune modulatory functions of MANF are likely to synergize to promote tissue recovery (Neves et al., 2016). We propose that the coordination of these functions is particularly important during regeneration, to promote the reparative phase of the regenerative process (Figure 1 see below). The effects of aging on MANF and CDNF expression and the consequences for organismal health are still under investigation.

### CDNF and Immune Cell Signaling

There is also evidence for regulation of inflammatory pathways by CDNF. However, the data is mostly restricted to brain resident immune cells – the microglia, and it is still unknown whether CDNF has a role in immune regulation outside the nervous system.

Primary microglia were shown to up-regulate CDNF in response to inflammatory stimuli, and CDNF supplementation attenuates the production of inflammatory cytokines (PGE2, Il-1β) in this cell type (Zhao et al., 2014). Unlike what was observed in NSCs after MANF stimulation (Zhu et al., 2016), the effects of CDNF were associated with inhibition of JNK pathways and not p38 (Zhao et al., 2014). In addition, CDNF overexpression in astrocytes is also sufficient to limit pro-inflammatory cytokine production (Il-6, Il-1β, and TNFα) (Cheng et al., 2013). In this context the effects were associated with inhibition of ER-stress (Cheng et al., 2013), a molecular pathway also affected by MANF (Apostolou et al., 2008) and generally associated with the regulation of inflammation (Grootjans et al., 2016). Thus it is possible that regulation of ER stress is also a mechanism involved in the immune modulatory function of MANF and CDNF.

The ability of CDNF to limit inflammation has also been observed *in vivo*, in murine models of 6-hydroxydopamine (6-OHDA) induced Parkinson’s disease (Nadella et al., 2014) or traumatic SCI (Zhao et al., 2016). In 6-OHDA treated rats, transient overexpression of hCDNF resulted in induction of endogenous CDNF, reduced neurotoxic glial cell activation and attenuated Il-6 levels in the substantia nigra (Nadella et al., 2014). In the SCI models, CDNF was delivered via bone marrow derived mesenchymal stem cells (BMSCs), engineered to produce CDNF. CDNF-BMSCs delivery improved nerve regeneration and motor recovery in this model and was associated with inhibition of neuroinflammation evaluated by reduced levels of several pro-inflammatory cytokines (PGE2, Il-1β, and TNFα) (Zhao et al., 2016).

While these studies demonstrate that inhibition of inflammation is observed *in vivo* in response to CDNF delivery, they do not elucidate whether this effect is merely a consequence of reduced neuronal damage or a mechanism involved in tissue recovery as shown for MANF. Moreover, it remains to be determined whether in an *in vivo* scenario, immune cells, such as microglia or recruited macrophages, mediate the effects of CDNF on neuroinflammation. While some studies suggest that CDNF can inhibit pro-inflammatory cytokine production by microglia (Zhao et al., 2014), other *in vitro* studies suggest that astrocyte-derived CDNF is not involved in preventing microglial activation (Rocha et al., 2012). Interestingly, delivery of Tregs, an immune cell type associated with inhibition of immune activation, in a 1-methyl-4-phenyl-1,2,3,6-tetrahydropyridine (MPTP)-damage model showed neuroprotective activities associated with induction of CDNF expression and modulation of microglial phenotypes (Reynolds et al., 2007). Thus, while this study suggests that CDNF expression in the brain can be modulated by immune cells (Tregs), it still remains to be determined whether CDNF itself modulates the effects observed on other immune cell types, like microglia.

## NTFs in Regeneration

Although NTFs can impact immune cell phenotypes in the context of tissue injury and repair in the CNS, evidence for their effects on regeneration through immune modulation is still limited. Nevertheless, some evidence suggests that NTFs can also impact the regenerative process by directly affecting stem cell function.

In skeletal muscle, neurotrophins and their receptors are expressed by different cell types and play an important role in tissue repair after injury. The low-affinity receptor for neurotrophins p75NTR is expressed in muscle progenitors (Mousavi and Jasmin, 2006) and regenerating myofibers (Colombo et al., 2013), and its activation is associated with pro-myogenic signaling (Colombo et al., 2011). Consistently, the inhibition of the NGF/p75NTR signaling pathway after skeletal muscle injury interferes with the normal process of myoblast fusion and delays muscle regeneration (Deponti et al., 2009). Although several p75NTR ligands are expressed in skeletal muscle (Sheard et al., 2002), BDNF in particular seems to play an important role in regulating satellite cell function and muscle regeneration. Mouse models in which BDNF was specifically depleted from skeletal muscle display reduced numbers of satellite cells and a temporary delay in the formation of new fibers after injury (Clow and Jasmin, 2010). Interestingly, the same study showed that the expression of BDNF, up-regulated in the early stages of regeneration, can also be found in infiltrating immune cells. BDNF-producing immune cells appear near p75NTR-positive regenerating myofibers (Colombo et al., 2013). Thus, it is possible that immune cell-derived BDNF can influence the regenerative process.

In parallel to findings in skeletal muscle regeneration, also in the skin multiple observations suggest a role for neurotrophins in tissue homeostasis and repair (Cheret et al., 2013). The levels of NGF are increased at skin wound sites (Matsuda et al., 1998), while in different mouse and rat models, the application of NGF to cutaneous wounds can accelerate the rate of wound healing (Li et al., 1980; Nithya et al., 2003; Shi et al., 2003). NGF is mostly expressed by proliferating keratinocytes (Marconi et al., 2003) and its expression and binding in human keratinocytes is necessary for the autocrine regulation of epidermal cell proliferation (Di Marco et al., 1993).

Finally, there is evidence for effects of GFLs in hematopoietic stem cells (HSCs) which express the GFL receptor ret. In HSCs GFL signaling promotes survival, expansion and *in vivo* transplantation efficiency (Fonseca-Pereira et al., 2014). GFLs are also important regulators of innate lymphoid cells in the intestine where they serve as damage sensors and regulate the production of Il-22, a regulator of gut inflammation (Ibiza et al., 2016) and intestinal stem cell function (Lindemans et al., 2015). However, a mechanistic link between regulation of immune cell phenotypes and stem cell function in the intestine is still to be determined.

## MANF and CDNF in Regeneration

The function of MANF and CDNF in regenerative contexts has only started to be explored. In the retina, we found that MANF supplementation improves regenerative success: congenitally blind mice that received transplanted photoreceptors supplemented with rMANF showed increase cell integration associated with accelerated and improved vision recovery, when compared to mice transplanted with the same photoreceptors without MANF supplementation (Neves et al., 2016). Several pieces of evidence suggest that these pro-reparative effects of MANF were mediated through immune modulation: (1) immune modulation is required for long-term survival of photoreceptors transplanted into the retina (West et al., 2010); (2) basal integration was impaired in mice with MANF-irresponsive macrophages (Neves et al., 2016); and (3) integration efficiency was not further enhanced by MANF when the same photoreceptors were transplanted into healthy mice, suggesting that the effects were not an enhancement of transplanted cell survival but rather the resolution of limitations in the host environment of a degenerating retina (Neves et al., 2016). Further experiments involving MANF supplementation in hosts depleted of macrophages or with MANF-irresponsive macrophages will be required to fully elucidate this cellular mechanism. Moreover, in mammals, it remains to be determined whether immune cells are indeed the source of MANF responsible for this physiological function. MANF expressing macrophages are associated with sites of cell integration (Neves et al., 2016), but experiments involving conditional ablation of MANF in immune cells have not been performed in the retina or any other physiological context of tissue injury. Interestingly, similar effects to those elicited by MANF have been observed in the same retinal regeneration paradigm after delivery of IGF-1 (West et al., 2012), a molecule with neurotrophic functions (Varela-Nieto et al., 2003) but also important immune modulatory properties (Johannesson et al., 2014; Tonkin et al., 2015). Importantly, immune regulation by IGF-1 is required for efficient regenerative success in the skeletal muscle (Tonkin et al., 2015) and also for neural protection in the degenerating retina (Arroba et al., 2011).

The regenerative paradigm of the retina can be used to test the effects of MANF in the reparative phases of the regenerative process, when newly formed cells need to functionally integrate in damage tissues. However, because endogenous stem cells do not participate in retinal regeneration in adult mice, this model does not elucidate if MANF can affect stem cell function. In other contexts, however, MANF was shown to impact stem cells directly: During embryonic development, MANF is intrinsically required in NSCs for neuronal migration and neurite outgrowth (Tseng et al., 2017). Adult NSCs also express MANF and intrinsic MANF signaling is necessary and sufficient for NSC differentiation and migration in ex-vivo explants, without affecting NSC proliferation and self-renewal (Tseng et al., 2018). These effects were associated with activation of ERK1/2 and STAT3 signaling. Importantly, in the context of ischemic stroke, NSCs participate in neural repair as a source of replacement neurons (Arvidsson et al., 2002). MANF delivery increased the number of newborn neurons found in the lesion area, by increasing cell migration but not NSC proliferation in the subventricular zone, an effect associated with functional recovery and not reproduced by other NTFs, such as GDNF (Tseng et al., 2018). It remains undetermined whether NSCs are the target of rMANF delivered to the brain in this context or if multiple cell types contribute to the effects observed. A recent report by the same group revealed that post-stroke delivery of MANF to the brain affected immune cell phenotypes, although no clear bias toward anti-inflammatory activation was detected (Matlik et al., 2018). Interestingly, the authors also found that loss of MANF in cells of neural origin had a negative impact on the size of the lesions with no effect on immune cell phenotypes (Matlik et al., 2018). While this study suggests that MANF derived from neural cells contributes to limit tissue damage in this model independently of the effects on immune cells, it also supports the notion that the effects on immune cells are due to an autocrine effect, as they are unaffected when MANF is ablated in non-immune cells of the nervous system. The authors did not address whether the effects observed in this study are related with the NSC functions described in the earlier study. One interesting hypothesis to consider in this context is whether the immune regulatory effects that MANF has on NSCs (Zhu et al., 2016), are part of the mechanism involve in recovery from stroke. Transplanted NSCs have been shown to improve recovery from different lesions in the CNS, including stroke, through mechanisms that go beyond cell replacement. NSC have immune modulatory properties and can affect immune cell phenotypes, a function that is required for their reparative functions in the CNS (Pluchino et al., 2005; Kokaia et al., 2012; Bernstock et al., 2017; Peruzzotti-Jametti et al., 2018).

## Conclusion

The studies described in this review open the possibility of MANF having important pro-regenerative effects *in vivo*, which may extend beyond the nervous system. The cytoprotective activity of MANF has been described in several cell types outside of the nervous system, including cardiomyocytes (Tadimalla et al., 2008; Glembotski et al., 2012) and pancreatic beta cells (Lindahl et al., 2014). Thus it is possible that it affects regenerative success in these systems and potentially also in other tissues where immune cells coordinate tissue regeneration.

**FIGURE 2:**
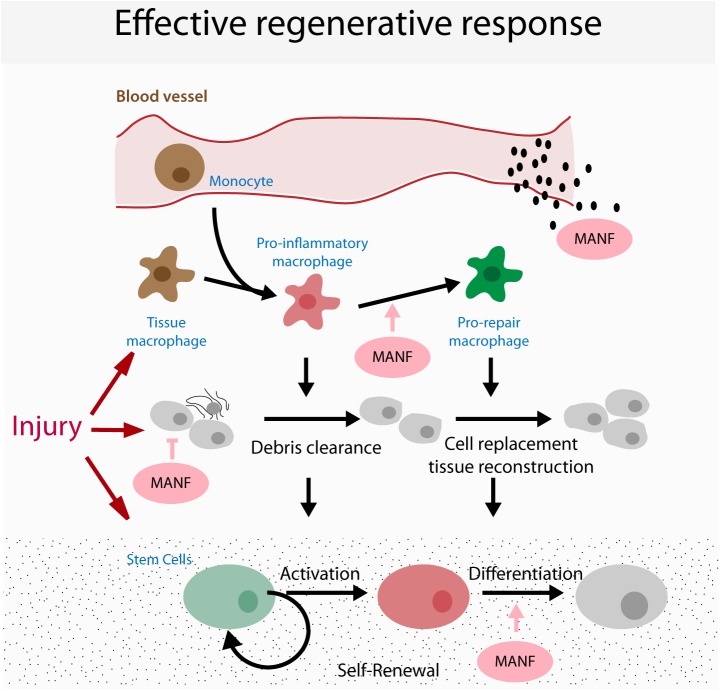
Model for possible targets of MANF in regeneration. Cartoon represents the coordination of the different cellular processes occurring during a regenerative response, highlighting the potential targets that mediate the pro-regenerative functions of MANF.

Future studies will be required to determine the cellular mechanisms involved in the regulation of regeneration by MANF. Independent *in vitro* studies have demonstrated that MANF can influence the biological function of different cell types that participate in the regenerative response: (1) cytoprotection of damaged cells, (2) regulation of immune cells and (3) regulation of stem cell function (although this has only been demonstrated for NSCs and may be associated with its neurotrophic function; Tseng et al., 2017, 2018). Thus, when delivered *in vivo* in a regenerative context, MANF has the potential to influence multiple processes, yet the relative contribution of each of these for regenerative success remains undetermined (Figure 2). Addressing this problem will involve the conditional ablation of MANF signaling independently in each target cell to evaluate the effects on regenerative success. However, these experiments have remained elusive due to a poor understanding of the molecular mechanisms of MANF function, including a lack of knowledge regarding a MANF receptor responsible for signal transduction. It is also still unknown whether the molecular mechanism and receptor transducing systems responsible for MANF function in different cell types are similar.

Due to its immune modulatory functions, MANF pro-reparative effects may be particularly useful when applied to improve regenerative success in disease conditions or in aging, where inflammation is an important limiting factor (Neves et al., 2017), however how MANF signaling is affected by aging has not yet been elucidated. Because the mechanisms governing tissue damage and inflammation in aging and disease are evolutionarily conserved (Neves et al., 2015) and diverse MANF functions are also conserved in invertebrate organisms (Palgi et al., 2009; Neves et al., 2016; Bai et al., 2018), these model systems could be used in the future to elucidate the molecular mechanism underlying cell-type specific MANF functions.

## Author Contributions

JN and PSV conceived and wrote the manuscript with input from HJ.

## Conflict of Interest Statement

HJ is employed by Genentech, Inc. The remaining authors declare that the research was conducted in the absence of any commercial or financial relationships that could be construed as a potential conflict of interest.
